# The Effects of Grain Boundary Misorientation on the Mechanical Properties and Mechanism of Plastic Deformation of Ni/Ni_3_Al: A Molecular Dynamics Study

**DOI:** 10.3390/ma13245715

**Published:** 2020-12-15

**Authors:** Jun Ding, Sheng-Lai Zhang, Quan Tong, Lu-Sheng Wang, Xia Huang, Kun Song, Shi-Qing Lu

**Affiliations:** 1 College of Mechanical Engineering, Chongqing University of Technology, Banan, Chongqing 400054, China; ZhangSL18184763041@126.com (S.-L.Z.); tongq123456@126.com (Q.T.); songkunmail@cqut.edu.cn (K.S.); shiqing.lu@cqut.edu.cn (S.-Q.L.); 2 School of Material Science and Engineering, Hefei University of Technology, Hefei 230009, China; wangls@mail.hfut.edu.cn

**Keywords:** Ni/Ni_3_Al interface, molecular dynamics, misorientation, mechanism of plastic deformation

## Abstract

The effects of grain boundary misorientation angle (*θ*) on mechanical properties and the mechanism of plastic deformation of the Ni/Ni_3_Al interface under tensile loading were investigated using molecular dynamics simulations. The results show that the space lattice arrangement at the interface is dependent on grain boundary misorientations, while the interfacial energy is dependent on the arrangement. The interfacial energy varies in a W pattern as the grain boundary misorientation increases from 0° to 90°. Specifically, the interfacial energy first decreases and then increases in both segments of 0–60° and 60–90°. The yield strength, elastic modulus, and mean flow stress decrease as the interfacial energy increases. The mechanism of plastic deformation varies as the grain boundary misorientation angle (*θ*) increases from 0° to 90°. When *θ* = 0°, the microscopic plastic deformation mechanisms of the Ni and Ni_3_Al layers are both dominated by stacking faults induced by Shockley dislocations. When *θ* = 30°, 60°, and 80°, the mechanisms of plastic deformation of the Ni and Ni_3_Al layers are the decomposition of stacking faults into twin grain boundaries caused by extended dislocations and the proliferation of stacking faults, respectively. When *θ* = 90°, the mechanisms of plastic deformation of both the Ni and Ni_3_Al layers are dominated by twinning area growth resulting from extended dislocations.

## 1. Introduction

Ni-based single-crystal superalloys exhibit excellent high-temperature creep strength, fatigue resistance, oxidation resistance, and thermal corrosion resistance due to their special two-phase microstructure, and are commonly used in the manufacturing of aero-engine turbine blades and other key components [1,2]. However, during the casting process of Ni-based superalloy blades, the large and low-angle grain boundaries caused by the differences in grain orientations can lead to scrapped castings. The difference in misorientation changes the free energy of the grain surface and the crystal microstructure [3], which results in the differences in mechanical properties and the scrapping of the castings. To avoid similar situations, it is necessary to carry out in-depth research on misorientation.

Many scholars have carried out research on misorientation through experiments. Zhao et al. [4], Shi et al. [5], and Huang et al. [6] have studied the effects of misorientation on the tensile properties, fracture properties, and grain boundary mechanical properties of the second-generation single-crystal superalloy DD6. Results show that the strength and stress rupture properties of superalloy DD6 decrease with increased misorientation. When the misorientation exceeds 10, the mechanical property of grain boundary significantly decreases. Li et al. [7] conducted creep testing on bicrystal specimens with low-angle grain boundaries. As observed by optical microscopy (OM) and scanning electron microscopy (SEM), when the temperature is high, the creep rupture life decreases as misorientation increases under a given set of test conditions. Tang et al. [8] studied the correlation between grain boundary misorientation and the precipitation behaviors of intergranular M_23_C_6_ carbides in a wrought Ni–Cr–W superalloy using EBSD. The result indicated that the misorientation angle can determine whether M_23_C_6_ precipitates or not. Alabbad et al. [9] studied the effects of grain boundary misorientation on the precipitation behavior of the η phase precipitates in the nickel-based alloy 718Plus. The results showed that the η phase precipitates in the form of thin lamellar precipitates at the low angle grain boundary. When the misorientation of the grain boundary is 40–50°, the *η* phase precipitates in the form of discrete precipitations.

The studies above show that misorientation affects many properties of nickel-based superalloys. However, these studies are mainly concentrated on macro-scale experimental observations, which can only observe the initial and final states of the material and cannot observe the microscopic evolution process and plastic deformation mechanism. Microscopic phenomena fundamentally affect the macro-mechanical properties of nickel-based superalloys. Therefore, it is necessary to understand how misorientation affects the properties of nickel-based superalloys at the atomic scale.

Molecular dynamics provide a good way to study the mechanical properties and microscopic mechanisms of plastic deformation for materials at the nanoscale. Some scholars have conducted studies using molecular dynamics simulations. Yashiro et al. [10] investigated dislocation nucleation and motion at the γ/γ′ interface in Ni-based superalloys. Zhu et al. [11] simulated the structure of a γ/γ′ phase interface in nickel-based superalloy. From the calculated results, they found that three dislocation network patterns, namely, square, rectangle, and equilateral triangle, appear on the {100}, {110}, and {111} interphase interfaces, respectively. Zhang et al. [12] evaluated the mechanical properties of the γ/γ interface using MD. Results show that the strengths of the configurations with true twin boundaries and those with ordered domain boundaries are almost the same, while the strength of a configuration with a pseudo-twin boundary is relatively low. Li et al. [13] investigated the influences of γ/γ interfaces on the tensile deformation behavior of γ-TiAl lamellae. Results in this work indicated that adjacent lamellae with a pseudo-twin or rotational boundary interface can constrain each other by in-plane stress due to modulus misfit; thus, the yield strength can be influenced.

Scholars have performed prominent research on the phase interface at the micro-scale, but those studies did not involve misorientation. The effect of misorientation on the phase interface has not been systematically studied. It is well known that the γ/γ’ phase interface affects the mechanical properties and plastic deformation of nickel-based superalloys [14,15,16,17,18,19,20,21,22]. Studies have shown that crystal orientation has a significant effect on the plastic deformation of nickel-based single-crystal superalloys [23], and the dislocation networks of the different phase interfaces show different degrees and patterns of damage under the same load and temperature [24]. These investigations indicate that the arrangement of the space lattice and crystal orientation at the interface are important for the mechanical properties and evolution of microscopic structures of nickel-based single-crystal superalloys. Therefore, molecular dynamics simulations have been used in this work to study the effects of misorientation on mechanical properties, such as yield strength, Young’s modulus, and flow stress of the Ni/Ni_3_Al interface from a microscopic perspective. By observing the microscopic structure evolution of the Ni/Ni_3_Al interface model under tensile loading, the influences of misorientation on the interfacial structure and microscopic mechanism of plastic deformation at the Ni/Ni_3_Al interface during tensile loading were investigated.

## 2. Modelling and Methods

**Figure 1:**
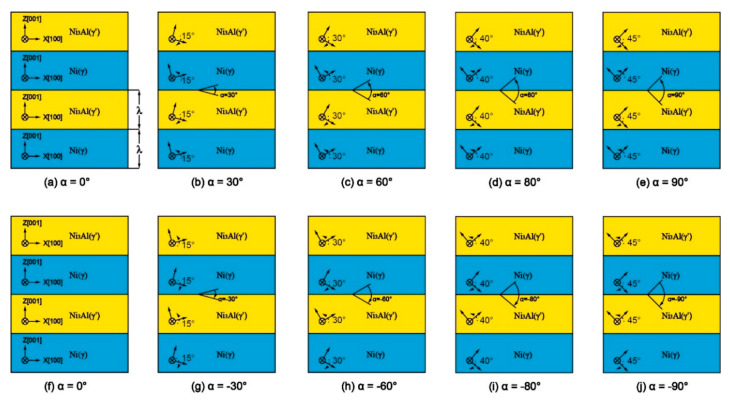
Models of Ni/Ni_3_Al multilayers (*λ* = 88.66 Å): (**a**) *α* = 0°; (**b**) *α* = 30°; (**c**) *α* = 60°; (**d**) *α* = 80°; (**e**) *α* = 90°; (**f**) *α* = 0°; (**g**) *α* = −30°; (**h**) *α* = −60°; (**i**) *α* = −80°; (**j**) *α* = −90°.

Using Atomsk–Version Beta 0.10.6 [25] to construct the 3D model of Ni/Ni_3_Al multilayers, the lattice constants of Ni (*a_γ_*) and Ni_3_Al (*a_γ_*_’_) used in this study were 3.52 Å and 3.573 Å, respectively. To reduce the residual stress caused by the lattice mismatch of the Ni (γ) and Ni_3_Al (γ’) phases, the sizes of the Ni/Ni_3_Al multilayers meet *na_γ_*_’_ = *ma_γ_*, where *n* and *m* are integers. Literature results show that the stress caused by the differences in the lattice parameters is relaxed within the range of the misoriented interface formed by 66 Ni_3_Al lattices and 67 Ni lattices [26], so *n* = 66 and *m* = 67 in this study. The model size in this study is about 236 Å × 72 Å × 354 Å, containing about 540,000 atoms. The model consists of an alternating stack of Ni and Ni_3_Al layers, where λ represents the thickness of a layer of Ni or a layer of Ni_3_Al in the nanoscale multilayer. In this study, the model was divided into four layers with *λ* = 88.66 Å, and the initial crystal orientations of the Ni layer and Ni_3_Al layer were x [100], y [010], and z [001]. The Ni/Ni3Al interface was unchanged (001), and the crystal directions of the Ni and Ni3Al layers were rotated with respect to the [010] direction axis so that the crystal directions of the Ni and Ni_3_Al layers were symmetrical with respect to the interface. The range of misorientation angle (*θ*) was 0–90°, and five angles were selected at *θ* = 0°, 30°, 60°, 80°, and 90°. *α* was defined as the angle which was rotated from the orientation of the Ni_3_Al layer to that of the Ni layer to distinguish the models with the same misorientation. For example, *α* is 90° in Figure 1e and *α* is −90° in Figure 1j even though the misorientation angles (*θ*) in both models (Figure 1e,j) are 90°. For the convenience of distinguishing between them, the following will use α to name each model, where the misorientation angle (*θ*) value is the absolute value of *α*. Since *α* = 0, the models in Figure 1a,f are the same.

LAMMPS 64–bit 20160902 [27] was used for molecular dynamics simulation in this study. The force field is described with an embedded-atom method (EAM) potential for a Ni–Al alloy system developed by Mishin et al. [28]. It has been proven that the EAM potential developed by Mishin et al. [28] can reproduce the elastic constants and stacking fault energies of Ni and Ni_3_Al. The periodic boundary conditions are used in three directions of the model, and the model was relaxed under the NPT system with a relaxation time of 50 ps before loading, enabling the system to achieve a stable state at its lowest energy. After relaxation, a strain rate of 10^9^ s^−1^ was applied in the z-direction of the model under the NPT system with a step size of 0.001 ps. The tensile loading lasted for 200,000 steps, and the final strain after loading was 0.2. The Nose-Hoover method [29,30] was used to adjust the temperature during the entire simulation. The entire simulation was performed at room temperature (300 K). In the end, the images and animations of the simulation process were reprocessed with the visualization software OVITO–2.9.0–win64 [31].

## 3. Results and Analysis

### 3.1. Mechanical Performance

**Figure 2:**
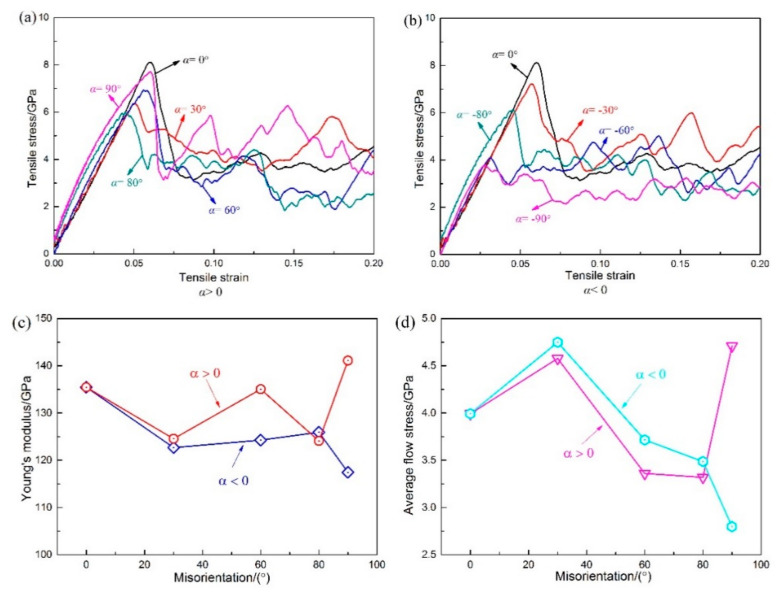
Stress–strain curves of models with *λ* = 88.66 Å: (**a**) α > 0, (**b**) α < 0; effect of misorientation on Young’s modulus and mean flow stress of Ni/Ni_3_Al multilayers: (**c**) Young’s modulus, (**d**) mean flow stress.

**Figure 3:**
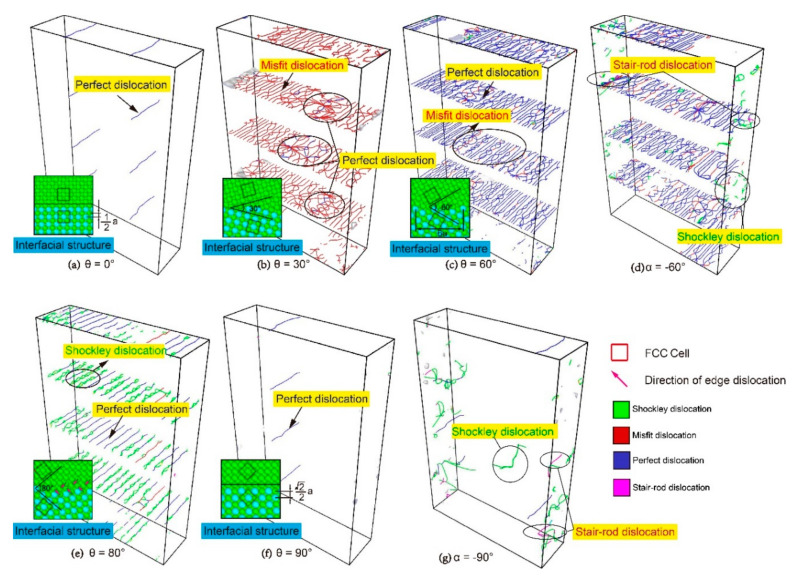
The dislocation analysis (DXA) before the yield point and the structure of the interface with different misorientations: (**a**) *θ* = 0°; (**b**) *θ* = 30°; (**c**) *θ* = 60°; (**d**) *θ* = −60°; (**e**) *θ* = 80°; (**f**) *α* = 90°; (**g**) *α* = −90°.

To investigate the effects of misorientation on the mechanical properties of Ni/Ni_3_Al multilayers, the stress–strain curve of each model was obtained (Figure 2a,b). As observed in Figure 2a,b, all models with *α* > 0 enter a plastic deformation stage after the tensile stress increases to a certain peak (yield strength), and then it decreases linearly with increasing strain. However, in the models with *α* = −60° and *α* = −90°, the stress–strain curves do not suddenly decrease after the stresses reach the yield point (Figure 2b). The reason is that the crystal orientations of the Ni and Ni_3_Al layers are different, although the models with the same misorientation have the same arrangement of space lattice in the case of *α* > 0 and *α* < 0. The difference affects the mechanical properties, resulting in a gap of Young′s modulus and mean flow stress between models with *α* > 0 and *α* < 0 (Figure 2c,d). The Young’s modulus slightly differs at *θ* = 30° and 80°, but more obviously so at *θ* = 60° and 90°. The difference in mean flow stress is small at *θ* = 30° and 80°, significantly increases at *θ* = 60°, and reaches a maximum at *θ* = 90 °. This is caused by the anisotropy of the Ni/Ni_3_Al multilayers 23. It can be seen from Figure 3d,g that Shockley dislocation and stair-rod dislocation appear in the models of α = −60 and −90 before the yield point. These dislocations are dispersed in the interface and layers; therefore, the densities of dislocations in the α = −60 and −90 models exceed those in the α = 60 and 90 models. Multiple dislocation emission points in the interface and layers lead to a decrease in Young′s modulus, which makes the model yield earlier and the yield strength decrease more greatly.

As shown in Figure 2a,b, for models with different misorientation angles (*θ*), the yield strength and changes in stress at the stage of plastic deformation are also different, and the relationship between these changes and the misorientation angle (*θ*) is nonlinear. The interface plays a key role in the yielding of Ni/Ni_3_Al multilayers. The change of yield strength is caused by differences in the interfacial structure as a result of increasing misorientation. When the misorientation angle (*θ*) = 0°, the Ni layer and Ni_3_Al layer have the same crystal orientations, the arrangement of the space lattice is also the same, the interfacial energy is the lowest, and there are only two perfect dislocations on the interface (Figure 3a). Therefore, the yield strength is the highest. When the misorientation angle (*θ*) = 90°, there is only one perfect dislocation (Figure 3f) on the interface. However, the distance between the two rows of atoms in the Z direction increases (Figure 3a,f), resulting in increased interfacial energy. Hence, its yield strength is lower than that of the model with *θ* = 0°. The stress–strain curve of the model with *θ* = 0° is similar to that of the Ni/NiAl(100) interface in the work of Hocker et al. [32], and the stress–strain curve of the model with *θ* = 90° is similar to those of the Ni/NiAl(110) interface and Ni/NiAl(111) interface in their study. When the misorientation angle (*θ*) = 60°, the arrangements of the Ni layer and Ni_3_Al layer in the model are different. The length of the constitutional unit of the interface is six lattice constants, the interfacial energy increases, and a large number of dislocations are formed at the interface. Most of these dislocations are perfect dislocations, and the rest are misfit dislocations (Figure 3c). Many nucleation sites on the dislocation network provide the ability for dislocation emission, so its yield strength is lower than that of the model with *θ* = 90°. When the misorientation angle (*θ*) = 30°, the length of the constitutional unit for the interface is 10.44 lattice constants, which means that the interfacial energy is higher than that of the model with *θ* = 60°. As shown in Figure 3b, the misfit dislocations are the main dislocations in the dislocation network, and the number of perfect dislocations is very small, which makes the yield strength lower than that of the model with *θ* = 60°. When the misorientation angle (*θ*) = 80°, the length of the constitutional unit for the interface increases to 32.65 lattice constants (Figure 3e), and the interfacial energy is higher than that of the model with *θ* = 30°. The interface can be regarded as the formation of a large number of edge dislocations, on which there are perfect dislocations arranged in parallel, resulting in a further decrease in the yield strength than that of the model with *θ* = 30°.

### 3.2. Plastic Deformation Mechanism

During the process of metal plastic deformation, the pattern of dislocation expansion depends largely on the number of active slip systems, and the Schmid factor is an important indicator to measure the active level of slip systems. By observing the plastic deformation mechanism of each model, we found that the misorientation angle (*θ*) is the main factor affecting the plastic deformation mechanisms of Ni/Ni_3_Al multilayers. To explore the influence of misorientation on the plastic deformation mechanism, we took five angles of α > 0 as the research object and calculated the Schmid factor (Table 1 and Table 2) of each slip system of the Ni and Ni_3_Al layers in these models to illustrate the influence of misorientation on the activity of the Ni/Ni_3_Al multilayer slip systems. The biggest Schmid factors of the slip system for both Ni and Ni_3_Al layer with misorientation of *θ* = 0°, 30°, 60°, 80° and 90° are 0.4082, 0.4830, 0.4830, 0.4406 and 0.4082, respectively, which represents for the most active slip system, as seen in Table 1 and Table 2.

When the misorientation angle (*θ*) = 0°, it can be found that the activated slip systems of the Ni and Ni_3_Al layers are the same, which are eight slip systems in the four slip planes of (11$\bar{1}$), (111), ($\bar{1\;}$1$\bar{1}$), and ($\bar{1\;}$11). When the misorientation angles (*θ*) = 30°, 60°, and 80°, all slip systems in the Ni and Ni_3_Al layers are activated. The Schmid factors of (111) [$0\bar{1}1$], ($\bar{1}$1$\bar{1}$) [011] in the Ni layer and (11$\bar{1}$) [011], ($\bar{1}11$) [$0\bar{1}1$] in the Ni_3_Al layer are always the largest under the same misorientation, and these four slip systems are always the most active during increased misorientation. When the misorientation angle (*θ*) increases to 90°, there are only four slip systems left in the Ni and Ni_3_Al layers, and the Schmid factors of these slip systems are the same. In the Ni layer, as the misorientation increases, the Schmid factors of four slip systems in the slip planes of (11$\bar{1}$) and ($\bar{1}11$) and two slip systems in the slip planes of (111) and ($\bar{1}$1$\bar{1}$) continuously decrease to zero, and the same phenomenon can also be observed in the Ni_3_Al layer. This shows that within the scope of the study, misorientation is inversely related to the number of active slip systems and the level of activity of the slip system. As misorientation increases, the number of active slip systems decreases, and the activity of the slip system lowers. These changes make dislocation propagation more difficult, which causes changes in the plastic deformation mechanism.

#### 3.2.1. Under the Condition of *θ* = 0°

**Figure 4:**
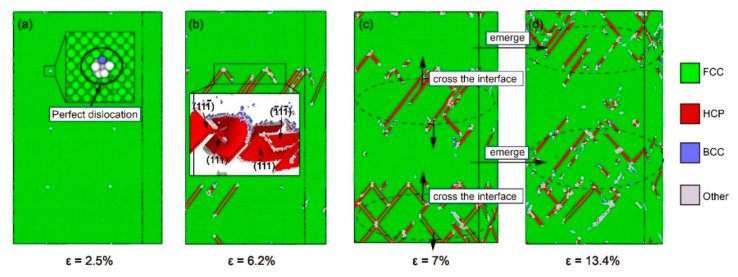
Common neighbor analysis (CNA) of the model with α = 0° under different strains *ε*: (**a**) *ε* = 2.5%, (**b**) *ε* = 6.2%, (**c**) *ε* = 7%, and (**d**) *ε* = 13.4%.

First, the deformation mechanism of Ni/Ni_3_Al multilayers at *θ* = 0° was analyzed. Figure 4a–d shows the common neighbor analysis (CNA) of dislocation under different strains. As shown in Figure 4a, when ε = 2.5%, two pre-existing perfect dislocations 1/2<110> are found at the interface of each layer of Ni/Ni_3_Al multilayers, which is caused by the difference in lattice parameters between the Ni and Ni_3_Al layers. When *θ* = 0°, the eight slip systems of (11$\bar{1}$), (111), ($\bar{1}$1$\bar{1}$), and ($\bar{1}$11) slip planes in the Ni layer are activated. Since the included angles between these slip systems and the direction of tensile loading are the same with each other, the Schmid factors of these eight slip systems are the same. However, the four slip systems (111) [$\bar{1}10$], (11$\bar{1}$) [$1\bar{1}0$], ($\bar{1}11$) [110], and ($\bar{1}$1$\bar{1}$) [110] were not activated because they are perpendicular to the direction of tensile loading (Table 1). All the Schmid factors are the same, meaning that dislocation can be simultaneously activated in these eight slip systems. Therefore, when the strain value is 6.2%, it can be observed from Figure 4b that Shockley dislocations have spread along four slip planes, (11$\bar{1}$), (111), ($\bar{1}$1$\bar{1}$), and ($\bar{1\;}$11), in the Ni layer, resulting in the formation of stacking faults in all four slip planes. The same phenomenon was found in the study of the Ni/NiAl interface by Hocker et al. [32]. When the tensile stress drops from the yield point to the lowest, the stacking faults have filled the Ni layer, and the interface acts as a barrier to prevent the Shockley dislocations from propagating to the Ni_3_Al layer, resulting in increased stress. However, as shown in Figure 4c, because of the low interfacial energy at the misorientation angle (*θ*) = 0°, some Shockley dislocations already cross the interface and enter the Ni_3_Al layer when *ε* = 9%. As the strain increases to *ε* = 13.4%, many stacking faults appear in the Ni_3_Al layer, the stacking faults in the Ni layer decrease significantly, and Shockley dislocations mainly spread in the Ni_3_Al layer. Since the activated sliding systems in the Ni_3_Al layer are the same as those of the Ni layer at *θ* = 0°, the plastic deformation mechanism has not changed. Consistent with the description in the literature, the stacking faults caused by Shockley dislocation are the primary plastic deformation mechanisms of the Ni/Ni_3_Al model with *θ* = 0° [26].

#### 3.2.2. Under Conditions of *θ* = 30° and 60°

**Figure 5:**
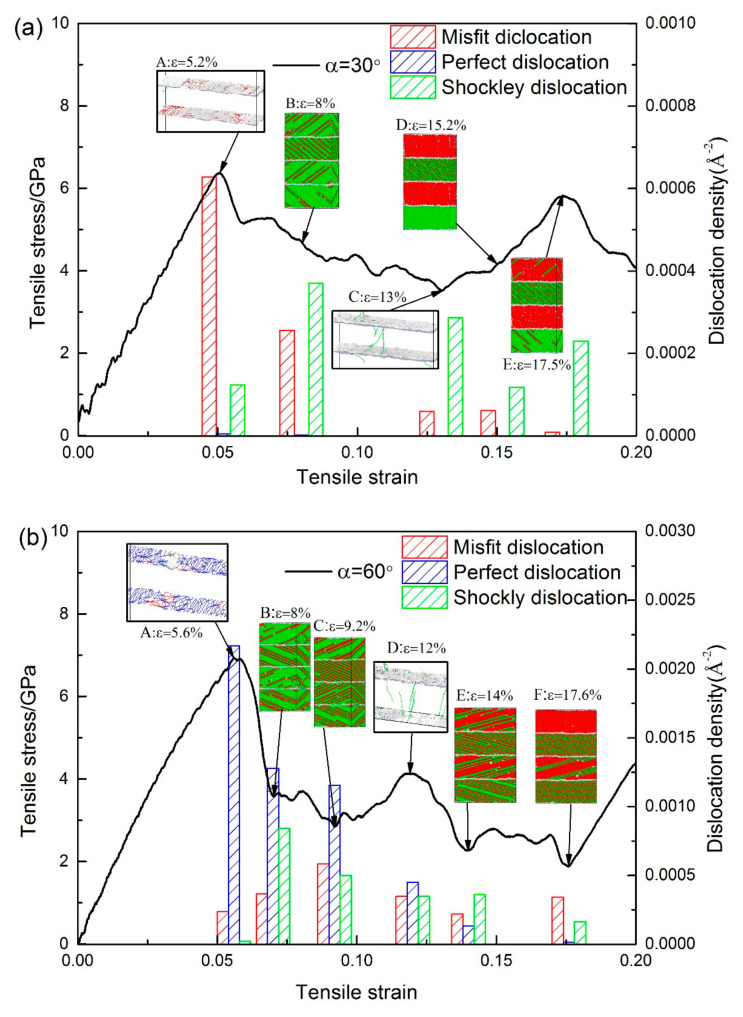
(**a**) Plots for both the stress-strain curve and dislocation density for the models with *α* = 30°. Snapshots A and C: DXA at *ε* = 5.2% and 13%, respectively. Snapshots B, D, and E: CNA at *ε* = 8%, 15.2%, and 17.5%, respectively. (**b**) The plots for both the stress-strain curve and dislocation density for the models while *α* = 60°. Snapshots A and D: DXA at *ε* = 5.6% and 12%, respectively. Snapshots B, C, E, and F: CNA at *ε* = 8%, 9.2%, 14%, and 17.6%, respectively.

There is a distinct difference in the stress–strain curves between the models with *θ* = 30° and *θ* = 60° (Figure 2a). To explore the reasons for this change, the stress–strain curve, a column diagram of dislocation density, common neighbor analysis (CNA), and dislocation analysis (DXA) under different strains of the model with *α* = 30° and *α* = 60° were studied (Figure 5a,b).

**Figure 6:**
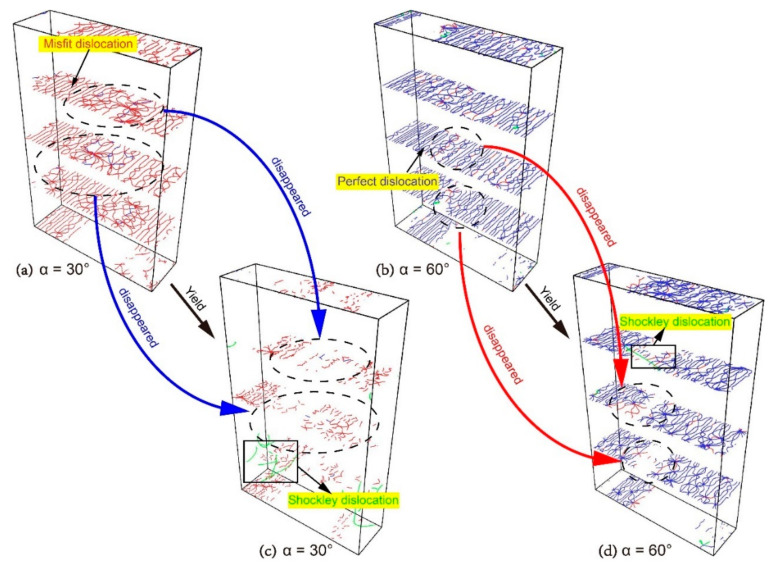
The dislocation analysis (DXA) of the models with different α before yield: (**a**) *α* = 30° and (**b**) *α* = 60°; and the dislocation analysis (DXA) of the models with different α during yielding: (**c**) *α* = 30° and (**d**) *α* = 60°.

The model with *α* = 30° yields at *ε* = 5.05%. According to the dislocation analysis (DXA) before and after the yield (Figure 6a,c), many misfit dislocations at the interface disappeared during yielding, and the Shockley dislocations appeared in the bottom Ni layer. The model with *α* = 60° yields at *ε* = 5.59%. It can be seen from the comparison of Figure 6b,d that the interface still retains a large number of dislocations and the Shockley dislocation appears in the Ni layer first. Comparing the DXA images of the two models before and during yielding, we can see that when *α* = 30°, the dislocations that accumulate on the interface are dominated by the misfit dislocations, which disappear in large numbers as the strain increases. The disappearance of dislocations leaves a large number of defects on the interface, reducing the strength yield and Young′s modulus of the model. For the model with *α* = 60°, most of the dislocations accumulated on the interface are perfect dislocations, which are less affected by the increase in strain. The dislocation network on the interface is largely preserved, resulting in higher yield strength and Young’s modulus than that of the model with *α* = 30° (Figure 2a,c).

Misfit dislocations on the interface of the model with α = 30° decreased consistently during tensile loading. According to the DXA at *ε* = 5.2% (Figure 5aA) and *ε* = 13% (Figure 5aC), the defects increase while the dislocations on the interface decrease. At the same time, the number of Shockley dislocations in the Ni and Ni_3_Al layers gradually increased after yielding. The factors mentioned above lead to stress decreases in the AC segment. In the CE segment, the dislocations on the interface disappear during the phase transition process. In addition, phase transition also leads to a massive disappearance of dislocation in the Ni_3_Al layer. The decrease of dislocation caused the rise of stress. According to the CNA at *ε* = 8% (Figure 5aB), *ε* = 15.2% (Figure 5aD), and *ε* = 17.5% (Figure 5aE), dislocations appearing in the Ni_3_Al layer first spread along the ($\bar{1}11$) plane and led to the proliferation of stacking faults at *ε* = 8%. Then the atomic configuration of Ni_3_Al layer changes from face–centered cubic (FCC) to hexagonal close–packed(HCP), and the Ni_3_Al layer undergoes a phase transition process. The dislocation disappears in the bottom Ni layer, and twin boundaries appear in the middle Ni layer at *ε* = 15.2%. Finally, some of the atoms change back to the FCC atomic configuration in the Ni_3_Al layer and the dislocation reappears in the bottom Ni layer at *ε* = 17.5%. As observed, the decomposition of the stacking fault into the twin boundary in the Ni layer caused by extended dislocation and the proliferation of the stacking fault in the Ni_3_Al layer are the primary plastic deformation mechanisms dominating the process of plastic deformation in the model.

The dislocation on the interface of the model with *α* = 60° is dominated by perfect dislocations. During tensile loading, the density of perfect dislocations on the interface has decreased, while the misfit dislocations and the Shockley dislocations in the Ni and Ni_3_Al layer have both increased, which leads to the drop of stress from *ε* = 5.6% (Figure 5b A) to *ε* = 9.2% (Figure 5b C). The curve between ε = 5.6% and *ε* = 8% (Figure 5b B) decreases at a faster rate due to the rapid increase of Shockley dislocations. The ($11\bar{1}$) and ($\bar{1}11$) planes in the Ni_3_Al layer have the slip systems with the biggest Schmid factors (Table 1 and Table 2), so the dislocation in the Ni_3_Al layer at ε = 8% spreads at both of the ($11\bar{1}$) and ($\bar{1}11$) planes simultaneously as a result of the spread of Shockley dislocations in the (111) plane and ($\bar{1}$1$\bar{1}$) plane of Ni layer. High interfacial energy and dense dislocation networks make the interface a strong barrier to the propagation of dislocation, so that the Shockley dislocations can only affect the emission of dislocation in the Ni3Al layer by reacting with dislocations on the interface, rather than passing through the interface. Based on the CNA, the dislocation in every Ni_3_Al layer only spreads along one slip plane when *ε* = 9.2% (Figure 5b C), which is the reason for the decreased Shockley dislocations. The reason for the increase of the curve from *ε* = 9.2% (Figure 5b C) to *ε* = 12% (Figure 5b D) is the significant reduction of the perfect dislocations at the interface. When the strain reaches 12% (Figure 5b D), the stress begins to decrease. The phase transition starts in the Ni_3_Al layer. The extended dislocation occurs in the Ni layer, and the stacking faults are decomposed into twin boundaries. When the strain increases to 14% (Figure 5b E), the number of Shockley dislocations in each layer reaches a stable value, the phase transition of the Ni_3_Al layers and the increase of twin boundaries continues. The curve begins to fluctuate near a value between 2 and 3 GPa. When *ε* = 17.6% (Figure 5b F), the phase transition in the top Ni_3_Al layer is finished and the structure of the Ni layer is stable. The phase transition of the middle Ni_3_Al layer continues. Finally, the dislocations decrease with the completed phase transition, causing increased stress after *ε* = 17.6%. Even though the plastic deformation processes are not the same for the model with *α* = 60° and the model with *α* = 30°, their primary plastic deformation mechanisms are the same.

As observed, the processes of plastic deformation for models with *α* = 30° and 60° are roughly divided into four stages: (i) the model yields after the elastic phase and the stacking faults appears in the Ni layer; (ii) as the strain increases, the stacking faults also appear in the Ni_3_Al layer; (iii) when the stacking faults fill the Ni_3_Al layer, the phase transition starts and the stacking fault begins to proliferate; (iv) when the atomic configuration of Ni_3_Al layer becomes HCP, the phase transition is completed.

**Figure 7:**
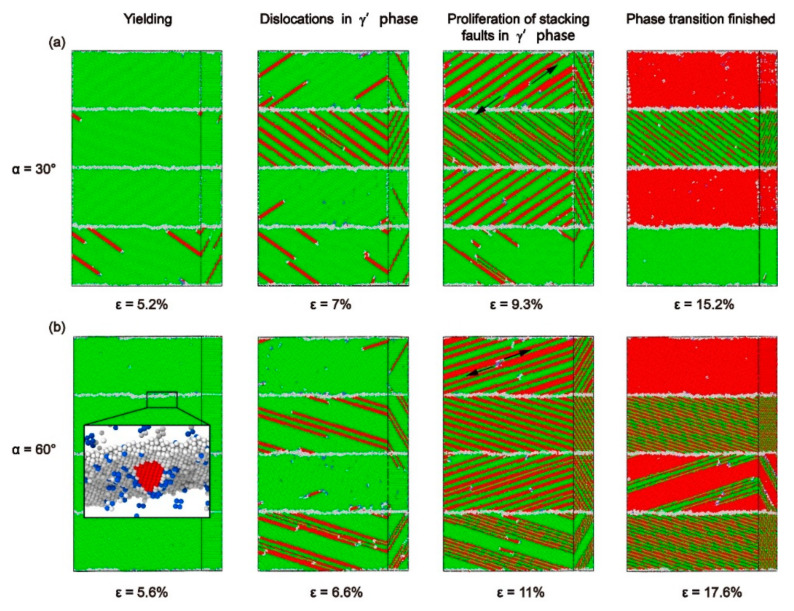
Plastic deformation processes of the model with different *α*: (**a**) *α* = 30° and (**b**) *α* = 60°.

The misorientation angle (*θ*) of the model with *α* = 30° is 30°, and all of the slip systems in the Ni and Ni_3_Al layers are activated. However, only the (111) [$0\bar{1}1$], ($\bar{1}$1$\bar{1}$) [011] in the Ni layer and (11$\bar{1}$) [011], ($\bar{1}11$) [$0\bar{1}1$] in the Ni_3_Al layer have the biggest Schmid factors (Table 1 and Table 2). In the first stage, when the model yields, dislocation first slides in the direction ($\bar{1}$1$\bar{1}$) [011], which has the maximum of the Schmid factor, and emits from the interface to the Ni layer. In the second stage, as the strain continues to increase, the spread of Shockley dislocations in Ni layer is blocked, and no other slip system is available, leading to an increase in the tensile stress (see Figure 6a). Subsequently, the conditions for dislocation emission in the Ni_3_Al layer are met, so Shockley dislocations slide in the direction ($\bar{1}11$) [$0\bar{1}1$], which has the maximum of the Schmid factor, and emit from the interface to the Ni_3_Al layer (Figure 7a). The emission of Shockley dislocations in the Ni_3_Al layer results in decreases of tensile stress (Figure 5a). When Shockley dislocations fill the Ni_3_Al layer, plastic deformation enters the third stage. The Ni and Ni_3_Al layers show different plastic deformation modes to cope with the increase of strain: The extended dislocation appears in the Ni layer and decomposes the original stacking faults into twin boundaries. Shockley dislocations in the Ni_3_Al layer continue to expand, which makes stacking faults proliferate (Figure 7a). In the fourth stage, the atomic configuration of the Ni_3_Al layer changes from FCC to HCP, and the phase transition is completed (Figure 7a). With the completion of the phase transition, the Shockley dislocations decrease, resulting in the increase of the tensile stress, such as the CE segment in Figure 5a. As shown in Figure 7b, the plastic deformation process of the model with *α* = 60° is approximately the same as that of the model with *α* = 30°, which indicates that the mechanism of microscopic plastic deformation remains unchanged after the misorientation angle (*θ*) increases from 30° to 60°.

Overall, when the misorientation angle (*θ*) = 30° and 60°, the interface is a strong barrier for the spread of Shockley dislocations. Ni and Ni_3_Al layers have different mechanisms of plastic deformation: the stacking faults caused by the propagation of Shockley dislocations in Ni layer will finally be decomposed into twin boundaries by extended dislocation while the expansion of Shockley dislocations in the Ni_3_Al layer leads to the proliferation of the stacking faults.

#### 3.2.3. Under Conditions of *θ* = 80°

**Figure 8:**
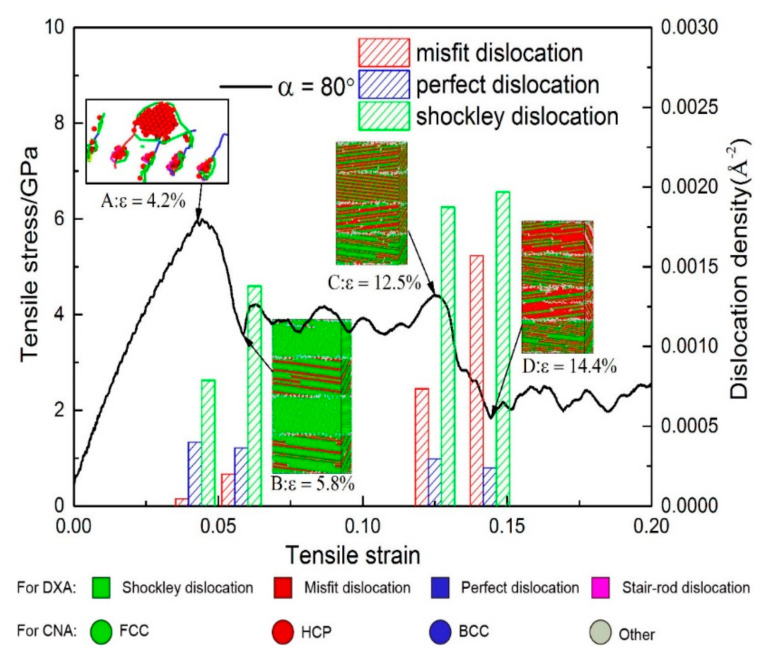
Plots for both the stress-strain curve and the dislocation density for the models with *α* = 80°. Snapshot (A): DXA at *ε* = 4.2%. Snapshots (B), (C), and (D): CNA at *ε* = 5.8%, 12.5%, and 14.4%, respectively.

Figure 8 shows a stress–strain curve of the model with *α* = 80°. Figure 8A shows the DXA at *ε* = 4.2%, which shows the Shockley partial dislocation loops emitted from the perfect dislocation at the interface. The model yields at *ε* = 4.4%, and then the stress fluctuates near 4 GPa. It is observed from the column diagram that the decreased stress is caused by the increased Shockley dislocation and misfit dislocation. Figure 8B shows that Shockley partial dislocation loops propagate in the Ni layer after yielding. As the strain increases, the densities of Shockley dislocation and misfit dislocation increase, while the density of perfect dislocation remains unchanged. When the strain reaches 12.5% (Figure 8C), the stress drops again and then fluctuates near 2 GPa. It is observed from Figure 8C,D that the slip system of the stacking faults in the Ni_3_Al layer changes. From the column diagram of dislocation density, it can be seen that the main reason for the drop of stress is the increase of misfit dislocation. The Young’s modulus and mean flow stress of this model are the lowest of the models with α > 0 (Figure 2c,d). As shown in Figure 3e, there are many perfect dislocations and Shockley dislocations on the interface of the model before yielding. The reason is that the arrangement of the space lattice near the interface of the model with *θ* = 80° is the same as that of the symmetric tilt grain boundary. This kind of interfacial structure can be seen as a dislocation wall made up of parallel edge dislocations, so the perfect dislocations on the interface are parallel to each other, rather than as messy as the model with *α* = 60°. Although the interfacial energy is the highest at *θ* = 80°, the barrier effect of the dislocation network on the spread of dislocation is greatly reduced due to the parallel perfect dislocations on the interface. Thus, the strength, Young′s modulus, and mean flow stress of the model with *θ* = 80° are the lowest of the five models.

**Figure 9:**
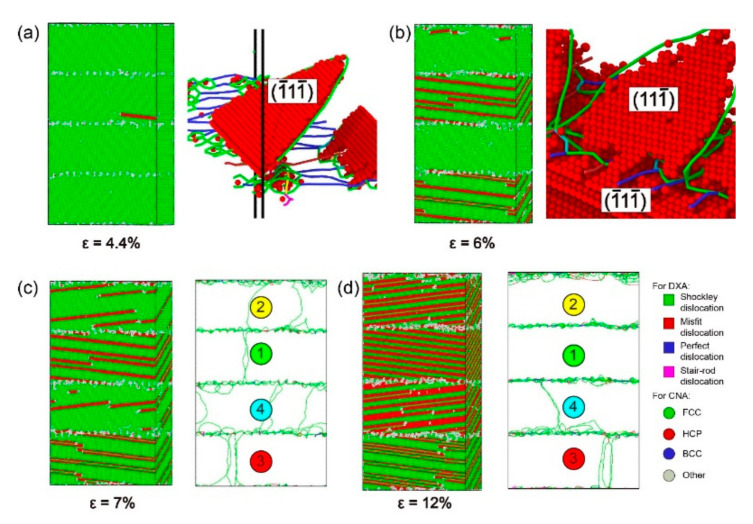
Common neighbor analysis (CNA) and dislocation analysis (DXA) of the model with *α* = 80° under different strains ε: (**a**) *ε* = 4.4%, (**b**) *ε* = 6%, (**c**) *ε* = 7%, and (**d**) *ε* = 12%.

**Figure 10:**
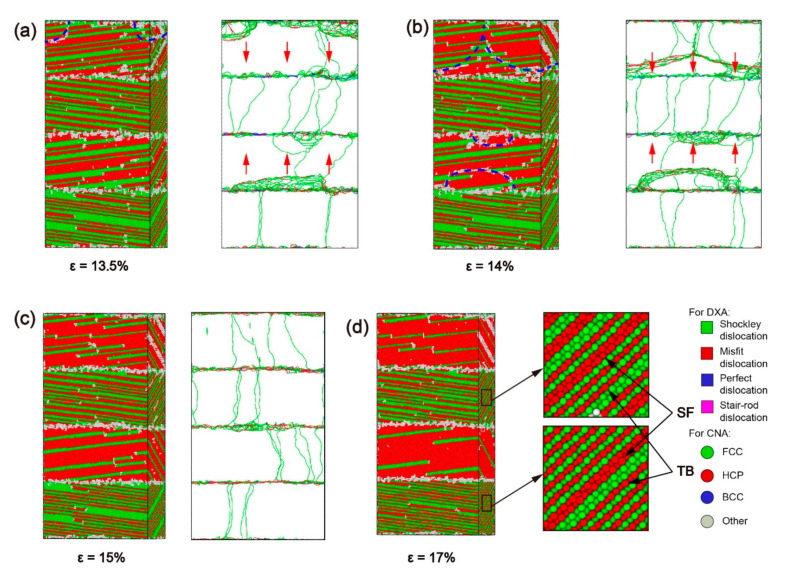
The common neighbor analysis (CNA) and dislocation analysis (DXA) of the model with α = 80° under different strains ε: (**a**) *ε* = 13.5%, (**b**) *ε* = 14%, (**c**) *ε* = 15%, and (**d**) *ε* = 17%.

To understand the mechanism of plastic deformation at *θ* = 80°, CNA and DXA under different strains for the model with *α* = 80° were studied (Figure 9 and Figure 10). When *ε* = 4.4%, the model has already yielded. Shockley dislocations emits from the perfect dislocations on the interface, spreading along the ($\bar{1}$1$\bar{1}$) plane to the Ni layer (Figure 9a). The reason is that the ($\bar{1}$1$\bar{1}$) [011] slip system is one of the slip systems with the biggest Schmid factors at *θ* = 80°. Therefore, the emission conditions of Shockley dislocations in this slip system are satisfied first. As the strain increases, Shockley dislocations in the Ni layer continuously propagate along with the slip system of ($\bar{1}$1$\bar{1}$) [011] and finally make contact with the interfacial dislocation networks. When *ε* = 6%, the model has already entered the stage of plastic deformation. The perfect dislocations on the interface are insufficient to form a barrier to the propagation of Shockley dislocations; Shockley dislocations go straight through the interface into the Ni_3_Al layer (Figure 9b). On the one hand, the (11$\bar{1}$) [011] slip system is one of the slip systems with the biggest Schmid factors in the Ni_3_Al layer at *θ* = 80° (Table 2), but on the other hand, the angle between the slip plane of Shockley dislocation in the Ni layer and that of the (11$\bar{1}$) [011] slip system is the lowest. Therefore, Shockley dislocations spread along the (11$\bar{1}$) [011] slip system on the (11$\bar{1}$) plane after entering the Ni_3_Al layer.

As shown in Figure 8, the stress between B and C fluctuates around 4 GPa. As observed from CNA in Figure 9c,d, when *ε* = 7%, stacking faults with the regular arrangement generate in the layers of ①②④ due to the spread of Shockley dislocations, and the stacking faults are separated into twin boundaries by extended dislocation in the layer of ③. When *ε* = 12%, the layers of ①②④ are full of stacking faults and the layer of ③ is full of twin boundaries, while the proliferation of stacking faults occurred in layer ④. From DXA in Figure 9c,d, when *ε* = 7%, there are fewer dislocations in the Ni and Ni_3_Al layers while Shockley dislocations accumulate at interfaces. When *ε* = 12%, the dislocations in the layers are further reduced. These reduced dislocations accumulate in the upper interface of the ② layer and the lower interface of the ④ layer. The Shockley dislocations at each interface are significantly denser at *ε* = 12% than at *ε* = 7%. Therefore, although dislocation increases with strain growth, the stress curve still oscillates around 4 GPa. As can be seen from the process of plastic deformation at *θ* = 80°, the barrier effect of the interface is significantly reduced so that the Shockley dislocations can pass through the interface directly, resulting in a large number of accumulated Shockley dislocations near the interface.

The curve suddenly drops at *ε* = 12.5%, and the stress fluctuates around 2 GPa after *ε* = 14.4%. The absorption capacity of the interface is insufficient to absorb the Shockley dislocations accumulated near the interface, and the accumulation of dislocations near the interface can be effectively reduced by changing the slip system of the Shockley dislocations via the movement of dislocation networks in the Ni_3_Al layer. According to the DXA in Figure 10a,b, the accumulated Shockley dislocations near the interface of the Ni_3_Al layer form a dislocation network and advance in the direction indicated by the red arrow in the Ni_3_Al layer. Additionally, the Ni_3_Al layer changes from single-crystalline to polycrystalline. With a blue dotted line as the boundary, the stacking fault in the area swept by the dislocation network spread along the ($\bar{1}11$) [011] slip system in the Ni_3_Al layer. Due to the generation of polycrystalline caused by the movement of the dislocation network, the Shockley dislocations in the Ni_3_Al layer dramatically increase, and the stress sharply declines.

When the dislocation network is absorbed by the interface in the forward direction, the Ni_3_Al layer changes from polycrystalline to single-crystal (Figure 10c), at which point the Shockley dislocations in the Ni_3_Al layer only spread along the ($\bar{1}11$) plane. Comparing the CNA in Figure 10c,d, it can be found that stacking faults proliferate in Ni_3_Al, and a phase transition occurs in the Ni_3_Al layer from the FCC structure to the HCP structure. The proliferation of stacking faults in the Ni_3_Al layer and the decomposition of stacking faults caused by extended dislocation in the Ni layer still dominate the plastic deformation of the model with *α* = 80°.

**Figure 11:**
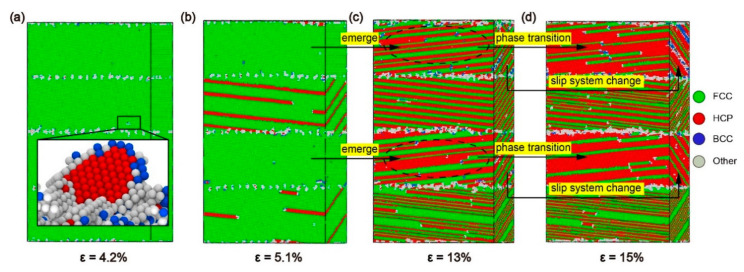
The common neighbor analysis (CNA) of the model with *α* = 80° under different strains ε: (**a**) *ε* = 4.2%, (**b**) *ε* = 5.1%, (**c**) *ε* = 13%, and (**d**) *ε* = 15%.

According to the process of plastic deformation for the model with *α* = 80°, it was found that the model of *θ* = 80° had gone through the same stages as the models of *θ* = 30°, 60°: (i) The model yields after the elastic phase, and the stacking fault first appears in the Ni layer; (ii) As the strain increases, the stacking fault also appears in the Ni_3_Al layer; (iii) After the stacking fault fills the Ni_3_Al layer, phase transition starts and the stacking fault proliferates; (iv) When the Ni_3_Al layer becomes an HCP structure, phase transition is complete (Figure 11). However, the plastic deformation of the *θ* = 80° model is different from that of the *θ* = 30° and 60° models: With increased misorientation up to 80°, the interface is no longer a strong barrier to the dislocation progress, directly leading to the accumulation of Shockley dislocations at the interface. Then, the accumulation of Stockley dislocations leads to the formation and disappearance of polycrystallinity.

Overall, when the misorientation angle (*θ*) = 80°, the interface is no longer a barrier for the spread of Shockley dislocations, and the Ni and Ni_3_Al layers have different mechanisms of plastic deformation: the stacking faults caused by the propagation of Shockley dislocations in the Ni layer are decomposed into twin boundaries by the extended dislocations and the continuous expansion of Shockley dislocations in the Ni_3_Al layer leads to the proliferation of stacking faults.

#### 3.2.4. Under Conditions of *θ* = 90°

**Figure 12:**
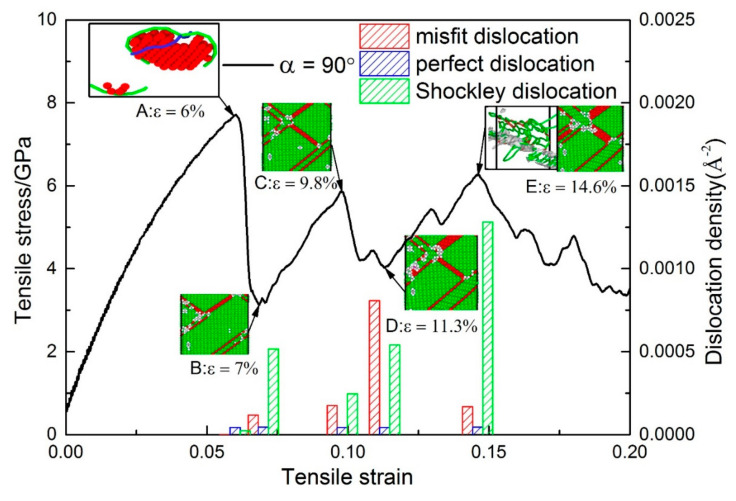
Plots for both the stress-strain curve and the dislocation density for the models while α = 90°. Snapshots (**A**) and (**E**): DXA at *ε* = 6% and 14.6%, respectively. Snapshots (**B**), (**C**), (**D**), and (**E**): CNA at *ε* = 7%, 9.8%, 11.3%, and 14.6%, respectively.

Figure 12 shows the stress–strain curve of the model with *α* = 90°. The model yields at 6% after the elastic phase, then the strain increases by only 1%, and the stress is reduced to 3 GPa (Figure 12). Figure 12A shows DXA at *ε* = 6.1%, and Shockley partial dislocation loops emitted from the perfect dislocation at the interface. It can be found from the column diagram of dislocation density that the increase of Shockley dislocations is the main reason for the decreased stress. With increasing strain, the tensile stress of the plastic deformation stage increases several times to around 6 GPa and then drops to about 4 GPa, exhibiting work hardening behavior, which is not the same as other models. In addition, work hardening also occurs in the Ni/NiAl interface with 110 and 111 interface orientation [32]. Figure 12B–E show CNA at *ε* = 7%, 9.8%, 11.3%, and 14.6% and DXA at *ε* = 14.6% in Ni_3_Al layer. It was found that the density of Shockley dislocation at *ε* = 9.8% is lower than that at *ε* = 7%. When the curve drops from the point of *ε* = 9.8% to *ε* = 11.3%, the density of misfit dislocation and Shockley dislocation greatly increases. When the curve rises from the point of *ε* = 11.3% to *ε* = 14.6%, the density of Shockley dislocation does not decrease, but increases further. It was found from CNA in Figure 12B,F that the proliferation of stacking fault and the growth of the twinning area occurred in the process of plastic deformation. It can be observed from DXA in Figure 12E that there are a number of Shockley dislocations at the intersection of stacking faults in the Ni_3_Al layer. The stacking Shockley dislocations form a dislocation network and strengthen the Ni_3_Al layer. The Young’s modulus and mean flow stress is the highest in all models with *α* > 0 (Figure 2c,d). As shown in Figure 3f, when the misorientation angle (*θ*) = 90°, since the space lattices of the Ni and Ni_3_Al layers are arranged in the same way, only one perfect dislocation exists in advance on each interface, so its interfacial energy is low, enabling the model of *α* = 90° to show high yield stress, Young′s modulus, and mean flow stress.

**Figure 13:**
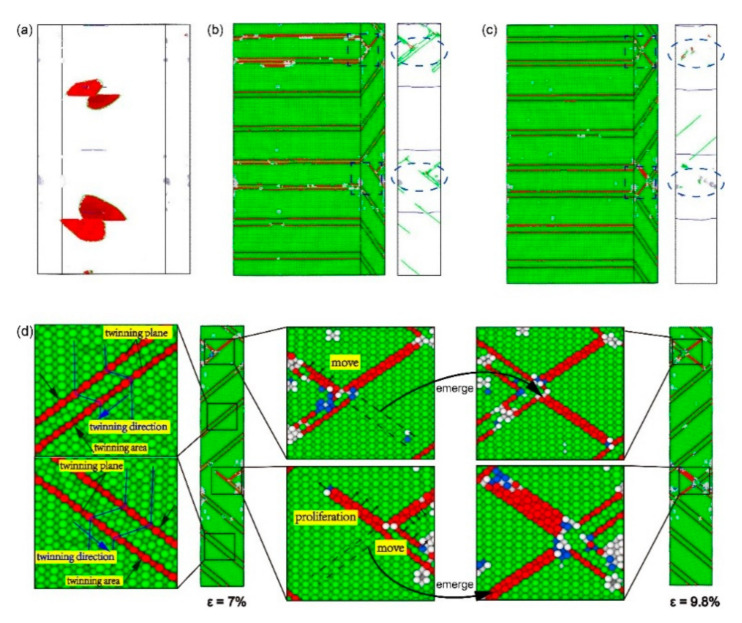
The common neighbor analysis (CNA) and dislocation analysis (DXA) of the model with *α* = 90° under different strains *ε*: (**a**) ε = 6.1%, (**b**) *ε* = 7%, and (**c**) *ε* = 9.8%; and (**d**) the microscopic structure of the model with *α* = 90° under *ε* = 7% and *ε* = 9.8%.

**Figure 14:**
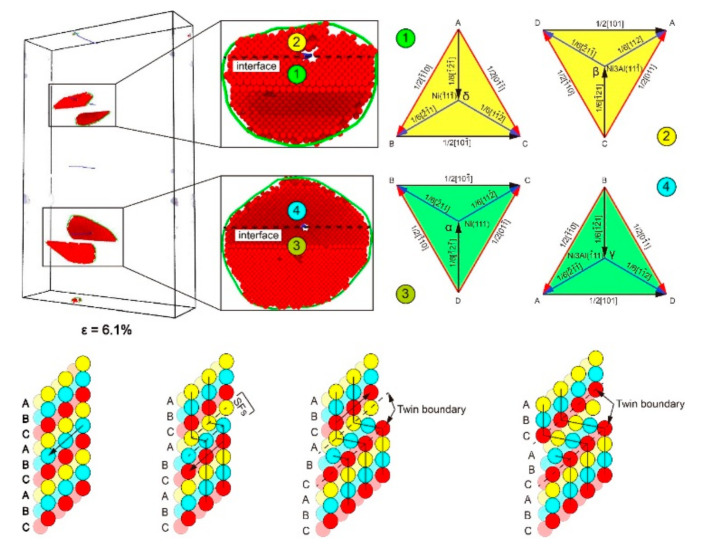
The slip system in dislocation and the forming process of the stacking fault and twin boundary in the model with *α* = 90°.

Figure 13 shows CNA of the model with *α* = 90°. When *ε* = 6.1% (Figure 13a), Shockley dislocations are centered on the perfect dislocations on the interface, extending to the Ni and Ni_3_Al layers simultaneously in the form of a dislocation loop, which is different from the priority emission of dislocation to the Ni layer observed in other models. The reasons for the difference are as follows: (i) the interface cannot prevent the extension of dislocation; (ii) after the increase in misorientation from 0° to 90°, there are two slip planes in the Ni and Ni_3_Al layers, respectively, rotating to the position parallel to the loading direction, and the number of activated slip systems decreases to four in two slip planes from eight in four slip planes of (11$\bar{1}$), (111), ($\bar{1}$1$\bar{1}$), ($\bar{1}$11) (Table 1 and Table 2); (iii) the slip planes of (111) and ($\bar{1}$1$\bar{1}$) in the Ni layer are parallel to the ($\bar{1}$11) and (11$\bar{1}$) planes in the Ni_3_Al layer, respectively, where the slip systems are parallel to each other. When the model yields, the slip systems of the ABC and BCD planes in the Ni layer coincide with the slip systems of the ACD and ABD planes in the Ni_3_Al layer, respectively (Figure 14). As an example, in the ABC plane, the Schmid factors of AB and AC are both 0.4082. Therefore, the two slip systems have the same activity level, which means that the component forces of the external force F in the AB and AC directions are the same, and the resultant force direction is the same as the δ direction. The perfect dislocations of AB and AC will be broken down into two Shockley partial dislocations due to the high energy costs:

(1) $$\mathrm{AB}\;\left(\mathrm{Perfect}\right){\;}_{\;\left(\delta \right)\;}\to A\delta \;\left(\mathrm{Shockley}\right){\;}_{\;\left(\delta \right)\;}+\delta B\;\left(\mathrm{Shockley}\right){\;}_{\;\left(\delta \right)\;}\;\mathrm{AC}\;\left(\mathrm{Perfect}\right){\;}_{\;\left(\delta \right)\;}\to A\delta \;\left(\mathrm{Shockley}\right){\;}_{\;\left(\delta \right)\;}+\delta C\;\left(\mathrm{Shockley}\right){\;}_{\;\left(\delta \right)\;}$$

or in vector form:

(2) $$1/2a{\left[\bar{1}\bar{1}0\right]}_{\;\left(\bar{1}1\bar{1}\right)\;}\to 1/6a{\left[\bar{1}\bar{2}\bar{1}\right]}_{\;\left(\bar{1}1\bar{1}\right)\;}+1/6a{\left[\bar{2}\bar{1}1\right]}_{\;\left(\bar{1}1\bar{1}\right)\;}\;1/2a{\left[0\bar{1}\bar{1}\right]}_{\;\left(\bar{1}1\bar{1}\right)\;}\to 1/6a{\left[\bar{1}\bar{2}\bar{1}\right]}_{\;\left(\bar{1}1\bar{1}\right)\;}+1/6a{\left[1\bar{1}\bar{2}\right]}_{\;\left(\bar{1}1\bar{1}\right)\;}$$

However, due to geometric and energy constraints,

(3) $$1/6a{\left[\bar{2}\bar{1}1\right]}_{\;\left(\bar{1}1\bar{1}\right)\;}+1/6a{\left[1\bar{1}\bar{2}\right]}_{\;\left(\bar{1}1\bar{1}\right)\;}\to 1/6a{\left[\bar{1}\bar{2}\bar{1}\right]}_{\;\left(\bar{1}1\bar{1}\right)\;}$$

At this time, it will pay a high energy cost to move a distance of Aδ again, so layer A only moves a distance of $1/6a{\left[\bar{1}\bar{2}\bar{1}\right]}_{\;\left(\bar{1}1\bar{1}\right)\;}$relative to layer B, result in the generation of stacking fault (Figure 14). The ACD, BCD, and ABD planes are similar to the ABC plane. Therefore, Shockley dislocations emit from the perfect dislocations on the interface and extend to the Ni and Ni_3_Al layer at the same time. The expansion of the dislocation loop causes the sudden increase of Shockley dislocations, and the stress of the model falls to around 3 GPa.

As shown in Figure 13b, when *ε* = 7% (Figure 12B), the Shockley dislocations extended along Ni ($\bar{1}$1$\bar{1}$)/Ni_3_Al (11$\bar{1}$) and Ni (111)/Ni_3_Al ($\bar{1}$11) encounter the stacking faults caused by each other, and the stacking fault acts as the barrier to more Shockley dislocations. At the same time, as the stress–strain curve drops from the point of *ε* = 6% to *ε* = 7%, the extended dislocation appears and expands in the Ni layer, separating the stacking faults into twin boundaries (Figure 13d). As the strain continues to grow, the same relative movement occurs between the crystal plane that forms the stacking faults and the adjacent FCC crystal plane, causing the stacking fault to separate into two twin boundaries (Figure 14). The dislocation line disappears from the Ni layer when the curve falls to point of *ε* = 7%. When *ε* = 9.8% (Figure 13c), the dislocation that meets in the Ni_3_Al layer disappears in large numbers, the reduction of dislocation makes the stress rise from point of *ε* = 7% to *ε* = 9.8%, and dislocation passes through the twin boundary that blocks its progress. The cross-stacking faults was also observed in the tensile behavior of the Ni/NiAl (110) interface [32].

A more detailed comparison of microscopic structural changes can be found in Figure 13d. During the tensile process from *ε* = 7% to *ε* = 9.8%, leading dislocation moves through the stacking faults and stops the spread of the extended dislocation on the crystal plane. When the “X”-pattern dislocation lock forms, the stacking faults and twin boundaries at the intersection block each other. The extended dislocation cannot break the stacking faults into twin boundaries after going through the intersection, but leads to the proliferation of the stacking faults with the growth of the twinning area behind the intersection. By observing the process of plastic deformation between *ε* = 6% and *ε* = 9.8%, the barrier effect of the interface on the extension of dislocations has completely disappeared at *θ* = 90°. However, due to the reduction of slip systems and the parallel relationship between the slip systems, the stacking faults become the new barrier to the dislocation extension. The stacking faults are more powerful than the interface as a barrier, causing the tensile stress of the model in plastic deformation to greatly fluctuate.

**Figure 15:**
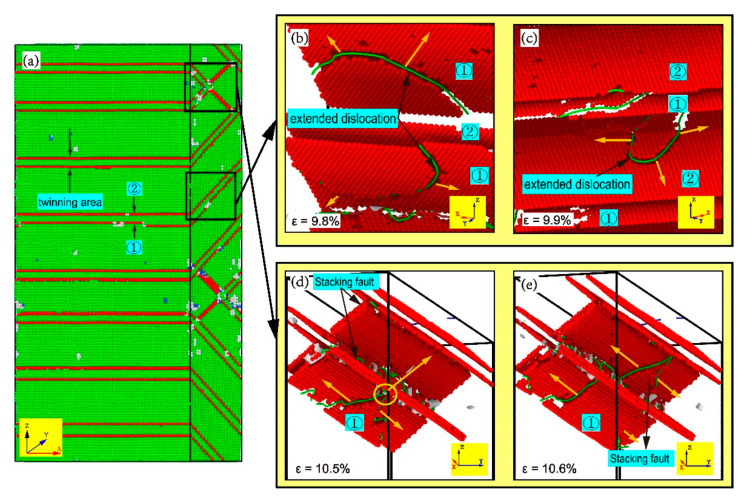
(**a**) The common neighbor analysis (CNA) of the model with *α* = 90° and dislocation analysis (DXA) of the model with *α* = 90° under different strain *ε*: (**b**) ε = 9.8%, (**c**) *ε* = 9.9%, (**d**) *ε* = 10.5%, and (**e**) *ε* = 10.6%.

Figure 15 is the CNA and DXA of stacking faults caused by extended dislocation under different strains between *ε* = 9.8% and *ε* = 14.6%. For clarity, the two twin boundary planes in the twinning area are marked as plane ① and plane ②, respectively. When the strain reaches 9.8%, the stress starts to fall again (Figure 12). As shown in Figure 15b, the extended dislocation loop has appeared in plane ① of the Ni layer, and the expansion of the extended dislocation loop causes the twin boundary to move down the distance of an atom. When *ε* = 9.9% (Figure 15c), the extended dislocation loop also appears in plane ② and the extension of the dislocation loop makes the twin boundary move up the distance of an atom. The growth of twinning area is caused by the relative slip between twin boundary and adjacent FCC crystal plane. Repetition of such processes during tensile loading makes the twinning area between twin boundaries grow. When *ε* = 10.5%, the microscopic structure of the Ni_3_Al layer is shown in Figure 15d. The reason for the short rise before *ε* = 11.3% is that the extended dislocation spreading to the Ni_3_Al layer is blocked by the dislocation network at the intersection of the stacking faults and twin boundaries. When *ε* = 10.6%, the extended dislocation passes through the dislocation network, resulting in the proliferation of the stacking faults and the drop of stress. When the curve drops to the point of *ε* = 14.6%, the growth of the twinning area and the proliferation of stacking faults is over. The ups and downs of the stress after the point of *ε* = 14.6% are the same as the deformation process between *ε* = 7% and *ε* = 14.6%. By analyzing the plastic deformation process between *ε* = 9.8% and *ε* = 14.6% in Figure 12, it was found that the primary mechanism of plastic deformation at *θ* = 90° is the growth of the twinning area and the proliferation of stacking faults caused by extended dislocation.

**Figure 16:**
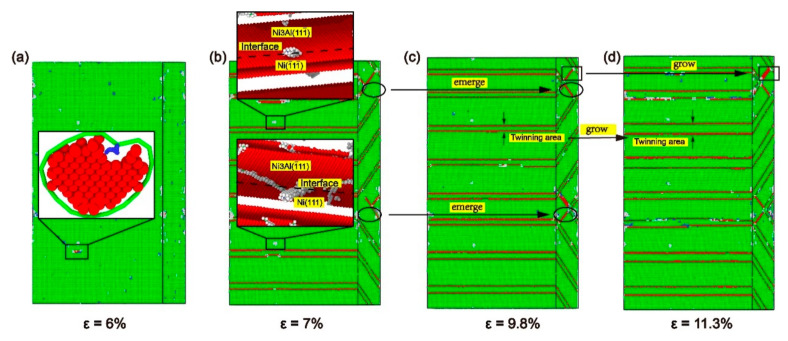
The common neighbor analysis (CNA) of the model with *α* = 90° under different strains *ε*: (**a**) *ε* = 6%, (**b**) *ε* = 7%, (**c**) *ε* = 9.8%, and (**d**) *ε* = 11.3%.

Figure 16 shows the primary process of plastic deformation for the model with *α* = 90°. The dislocation nucleates at the perfect dislocations on the interface and extends simultaneously in the Ni and Ni_3_Al layers, unobstructed by the interface (Figure 16a). As observed in Figure 16b, when the misorientation angle (*θ*) = 90°, the dislocations in the Ni and Ni_3_Al layers are located in different slip systems, but still in the same plane. When a dislocation extended along a pair of slip systems encounters a twin boundary on another pair of slip systems, it ends up passing through the twin boundary (Figure 16b,c). In addition, the growth of the twinning area and the proliferation of stacking faults can also be observed (Figure 16c,d).

Overall, when *θ* = 90°, the interface no longer hinders the expansion of dislocations, and stacking faults becomes the new barrier to the expansion of Shockley dislocations. The mechanism of plastic deformation for model with *θ* = 90° is the growth of the twinning area and the proliferation of stacking faults caused by extended dislocation, which is quite different from the mechanisms of plastic deformation for other models.

## 4. Conclusions

The mechanical properties of the interface depend on misorientation. Different misorientations have effects on the structure and interfacial energy of the interface, which leads to differences in yield strength, elastic modulus, and mean flow stress.

The difference in misorientation will change the mechanism of plastic deformation for the Ni/Ni_3_Al interface. When the grain boundary misorientation angle (*θ*) increases from 0° to 90°, the extension of dislocation changes. When *θ* = 0°, the plastic deformations of the Ni and Ni_3_Al layers are dominated by stacking faults caused by Shockley dislocation. When *θ* = 30°, 60°, and 80°, the mechanisms of plastic deformation for the Ni_3_Al and Ni layers are the proliferation of stacking faults and the decomposition of stacking faults caused by extended dislocation, respectively. When *θ* = 90°, the mechanisms of plastic deformation for both Ni and Ni_3_Al layers are dominated by the growth of the twinning area as a result of extended dislocation.

The difference in orientation influences the mechanical properties of the interface. Comparing the *α* = 60°, −60°, 90°, and −90° models, it was found that the yield strength, Young′s modulus, and mean flow stress of the model were significantly different when the crystal directions of the Ni and Ni_3_Al layers were interchanged without changing the arrangement of the space lattice in the model.

## Figures and Tables

**Table 1:** Schmid factors of different slip systems under different misorientations in the Ni layer.

Slip Plane	Slip Direction	0°	30°	60°	80°	90°
**(111)**	[$\bar{1}10$]	0	0.1294	0.2788	0.3697	0.4082
	[$10\bar{1}$]	0.4082	0.3536	0.2041	0.0709	0
	[$0\bar{1}1$]	0.4082	0.4830	0.4830	0.4406	0.4082
**(11** **$\bar{1}$** **)**	[$1\bar{1}0$]	0	0.0747	0.0747	0.0323	0
	[101]	0.4082	0.3536	0.2041	0.0709	0
	[011]	0.4082	0.2788	0.1294	0.0385	0
**(** **$\bar{1}11$** **)**	[110]	0	0.0747	0.0747	0.0323	0
	[101]	0.4082	0.3536	0.2041	0.0709	0
	[$0\bar{1}1$]	0.4082	0.2788	0.1294	0.0385	0
**(** **$\bar{1}$1 ** **$\bar{1}$** **)**	[110]	0	0.1294	0.2788	0.3697	0.4082
	[$\bar{1}01$]	0.4082	0.3536	0.2041	0.0709	0
	[011]	0.4082	0.4830	0.4830	0.4406	0.4082

**Table 2:** Schmid factors of different slip systems under different misorientations in the Ni_3_Al layer.

Slip Plane	Slip Direction	0°	30°	60°	80°	90°
(111)	[$\bar{1}10$]	0	0.0747	0.0747	0.0323	0
	[$10\bar{1}$]	0.4082	0.3536	0.2041	0.0709	0
	[$0\bar{1}1$]	0.4082	0.2788	0.1294	0.0385	0
(11$\bar{1}$)	[$1\bar{1}0$]	0	0.1294	0.2788	0.3697	0.4082
	[101]	0.4082	0.3536	0.2041	0.0709	0
	[011]	0.4082	0.4830	0.4830	0.4406	0.4082
($\bar{1}11$)	[110]	0	0.1294	0.2788	0.3697	0.4082
	[101]	0.4082	0.3536	0.2041	0.0709	0
	[$0\bar{1}1$]	0.4082	0.4830	0.4830	0.4406	0.4082
($\bar{1}$1$\bar{1}$)	[110]	0	0.0747	0.0747	0.0323	0
	[$\bar{1}01$]	0.4082	0.3536	0.2041	0.0709	0
	[011]	0.4082	0.2788	0.1294	0.0385	0
